# A Kinematic Model of a Humanoid Lower Limb Exoskeleton with Hydraulic Actuators

**DOI:** 10.3390/s20216116

**Published:** 2020-10-27

**Authors:** Sebastian Glowinski, Tomasz Krzyzynski, Aleksandra Bryndal, Igor Maciejewski

**Affiliations:** 1 Department of Mechanical Engineering, Koszalin University of Technology, 75453 Koszalin, Poland; tomasz.krzyzynski@tu.koszalin.pl (T.K.); igor.maciejewski@tu.koszalin.pl (I.M.); 2 Institute of Health Science, Slupsk Pomeranian University, 76200 Slupsk, Poland; olka-kulczyk@wp.pl

**Keywords:** inertial measurement unit, exoskeleton model, kinematic, hydraulic exoskeleton

## Abstract

Although it is well-established that exoskeletons as robots attached to the extremities of the human body increase their strength, limited studies presented a computer and mathematical model of a human leg hydraulic exoskeleton based on anthropometric data. This study aimed to examine lower limb joint angles during walking and running by using Inertial Measurement Units. The geometry and kinematic parameters were calculated. Twenty-six healthy adults participated in walking and running experiments. The geometric model of a human leg hydraulic exoskeleton was presented. Joint angle data acquired during experiments were used in the mathematical model. The position and velocity of exoskeleton actuators in each phase of movement were calculated using the MATLAB package (Matlab_R2017b, The MathWorks Company, Novi, MI, USA). The highest velocity of the knee actuator during walking and running was in the swing phase, 0.3 and 0.4 m/s, respectively. For the ankle and hip joints, the highest velocity of actuators occurred during the push-off phase. The results with 26 healthy subjects demonstrated that the system's compliance can be effectively adjusted while guiding the subjects walking in predefined trajectories. The developed mathematical model makes it possible to determine the position of lower limb segments and exoskeleton elements. The proposed model allows for calculating the position of the human leg and actuators’ characteristic points.

## 1. Introduction

In recent years, extensive research has been conducted around the world on wearable robots and orthotic devices that support the movement of lower limbs. Wearable robots are devices that constitute a new class of articulated mechanical systems. These particular types of robots operate in close contact with a human user. They are worn by an operator like a suit, and their kinematic structure is similar to a human limb. Wearable robots are called exoskeletons. Many researchers have investigated problems related to exoskeletons. In the article by Pons et al. [1], the subject of exoskeletons is widely presented, including biomechatronic design, cognitive and physical human-robot interactions, wearable robot technologies, kinematics, dynamics, and control. Many categories of devices are referred to as exoskeletons, e.g., devices that assist in a particular activity (such as climbing or moving heavy objects) or devices that assist in rehabilitation to help persons with disabilities regain a satisfactory level of motor fitness [2,3]. Exoskeletons are used in different fields such as military exoskeletons, general-purpose exoskeletons, or medical exoskeletons [4,5]. Exoskeletons are designed and constructed by taking into account the shape and functions of the supported parts of the human body. The main purpose of their use is to increase strength, speed, and performance. They are mainly used in the military, industry, and medicine, and their main application is carrying heavy loads [6,7]. They provide greater load carrying mobility with less load and allow the coverage of greater distances than present conditions allow in the field (infantry). Fire-fighters can use the exoskeleton to climb a large number of stairs and to climb great heights with heavy weights without getting tired. In medicine, they are used in the rehabilitation process. Exoskeletons support movement if the patient has severely weakened muscles and help them to perform it properly. They make it possible to steer movements in the correct trajectories and help patients to relearn the correct movement patterns. They give the ability to walk to patients with a total or partial loss of gait control [6]. Hydraulic, pneumatic, or electric systems are used as exoskeleton actuators [8,9]. The advantages and disadvantages of each system are described in detail in Reference [6]. Typical examples of lower limb exoskeletons are ReWalk (Marlborough, MA, USA) and HAL (The Hybrid Assistive Limb, Gauken-Minami, Tsukuba, Ibaraki Prefecture, Japan) [10].

One of the problems associated with exoskeletons is the misalignment between the user and robot. This problem concerns the knee joint in particular. Most constructors treat the knee joint as 1 DoF (Degree of Freedom) joint system. In order to compensate this problem, Lee et al. proposed a polycentric knee structure with a rotary encoder sensor [11].

However, for the exoskeleton to be able to perform its task, it is necessary to determine how it should work. One way is to implement the trajectories (angles) of individual joints during walking and running. This allows the selection of actuator parameters, like dimension and position in the function of human leg length [12].

Typical gait analysis methods use cameras with reflective markers to track body position. The optical system is successfully used in many research fields, including sports and medicine. However, it is limited to the laboratory workspace that the camera requires [13]. An alternative to this limitation is the use of body-attached inertial sensors that acquire acceleration and angular velocity data.

Inertia sensors are attracting interest constantly due to their miniature size and reduced power consumption. The cost of sensors is many times lower than that of an optical system [14,15]. In the article, we used the ProMove mini platform (Inertia Technology B.V., AG Enschede, The Netherlands) with wireless interactive sensors to calculate the joint angles [16]. A detailed description of the measurement and recording of parameters was included in previous works [7,17].

The aim of this research was to create a geometrical and mathematical model of the hydraulic exoskeleton. Hip, knee, and ankle gait angles were obtained using IMUs (Inertial Measurement Unit) implemented in the proposed model. In the first section of this paper, the experimental parameters of walking and running for the hip, knee, and ankle in the sagittal plane were calculated. Then, the basic model of the hydraulic exoskeleton in the sagittal plane is presented. Next, coordinates of the exoskeleton and human leg characteristic points as a mathematical explanation are established step by step. The angle values obtained during the experiment were implemented in the model. The change of the actuator lengths in the angle function was determined using the MATLAB package. Finally, a brief conclusion with limitations of the study is presented.

## 2. Materials and Methods

### 2.1. Lower Limb Angles during Walking and Running

**Figure 1:**
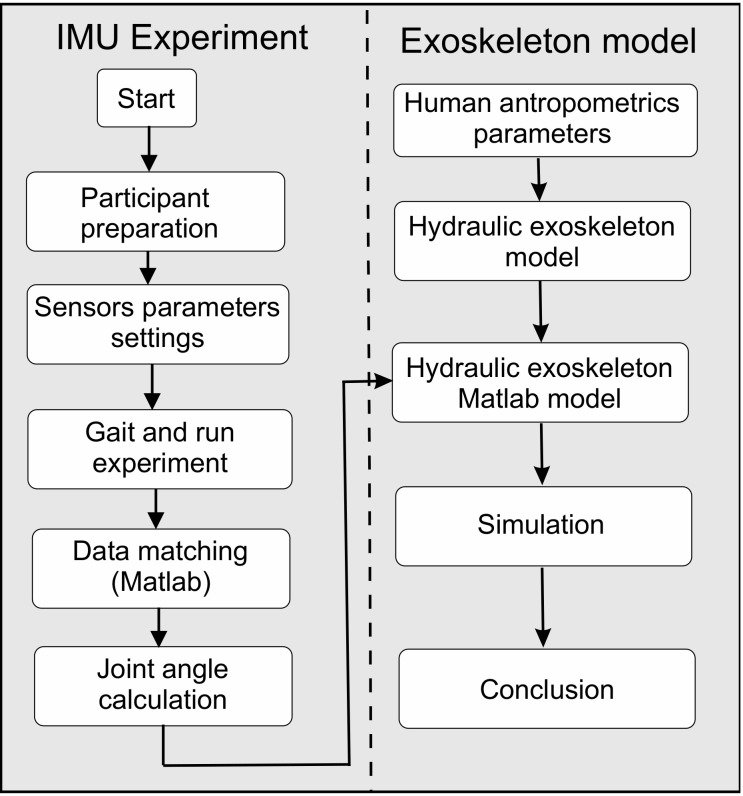
Flowchart of the experimental procedure.

Our research was divided into two parts: IMU experiment and exoskeleton model with the simulation (Figure 1). Twenty-six subjects (male = 14, female = 12) participated in this study. The study protocol was approved by the Bioethics Committee at the district medical chamber in Gdansk (KB-14/20). Before the study, the principal investigator explained all procedures in detail to the subjects. The acceptance rate was 100%. This means that all subjects agreed to wear the sensors and participate in the experiment. The participants’ mean age (SD) was 22.8 years (0.79), mean height was 169.45 cm (8.26), and mean body weight was 68.05 kg (9.27), respectively. All the tested persons walked and ran on a flat surface at the speed they preferred. The research equipment included a ProMove mini platform with mini sensors [17]. This device consists of a wireless network to transfer data to a computer. This makes it possible to analyze data in real time. By using ProMove mini sensors, it is possible to record data in continuous time. Each sensor has a built-in memory with storage up to 7 h [18]. The device contains triaxial accelerometers, gyroscopes, and magnetometers. All channels are sampled at 1000 Hz. The range of accelerometers is ±16 g and the range of the gyroscopes is to 2000 deg/s. The flash memory of each sensor is 2 GB, and the low-power RF (radio-frequency) transceiver is in the 2.4 GHz license-free band. Because of the ergonomic design, the ProMove sensors can be easily attached to the body with a strap. Each sensor is 51 to 46–15 mm in size. This weighs 20 g including the battery. Data collection <100 ns is synchronized by an Inertia gateway as the central hub connected to a personal computer via a USB (Universal Serial Bus). All data (experimental samples, orientation as Euler angles or quaternions, acceleration along three axes and angular velocities) are downloaded by the Inertia Studio package. Next, they are recorded for further analysis. During the experiment, sensors were placed along the right and left legs, more specifically on the thigh, lower leg, and foot. In a global coordinate system, the *X*-axis is the moving direction and the *Y*-axis is the lateral direction. The *Z*-axis presents the opposite direction of gravity. The orientation of each sensor is calculated relative to the Earth’s reference frame. As a result, roll, pitch and yaw sensor angles are acquired. Based on the orientation of each sensor, the angles in the individual joints of the lower limb in the sagittal plane were acquired. A joint coordinate system (JCS) was proposed by the Standardization and Terminology Committee (STC) of the International Society of Biomechanics [19]. In experiments, hip, knee, and ankle motions were calculated by the orientation of each segment. Next, these were described in the *XYZ*-Euler angle representation. IMUs were placed on the parallel plane to the anteroposterior plane. The proposed exoskeleton model works in the sagittal plane, and, therefore, we based our calculations only on the angles in the sagittal plane. To compute the hip, knee, and ankle angles, the sensor frames were transformed into a joint/mutual coordinate system (JCS). The multiplication of the single rotation matrices around each axis transforms the sensor frame at any time step into the initial sensor frame. The rotation matrices are determined by the equation below.

(1) $$R_{i}=\left[\begin{array}{ccc} c\theta c\psi & -c\theta s\psi & s\theta \\ c\phi s\psi +s\phi s\theta c\psi & c\phi c\psi -s\phi s\theta s\psi & -s\phi c\theta \\ s\phi s\psi -c\phi s\theta c\psi & s\phi c\psi +c\phi s\theta s\psi & c\phi c\theta \end{array}\right]$$

where c and s denote the cos and sin function and *ϕ*, *θ,* and *ψ* are the rotation angles about *U*–, *V*–, and *W*–axes, respectively.

Unfortunately, as with all IMU sensors, the ProMove mini sensors also have problems with a random bias that builds up over time. Accurately determining the body position and orientation for more than a few seconds is impossible because of a growing drift. To overcome this problem, some researchers use accelerometers and magnetometers in a complementary filter [20,21]. In addition, tracking algorithms that restrict limb movement are often used. This procedure reduces the effect of sensor drift on orientation and position estimates [22]. Favre et al. based their research on two sensor modules installed on the lower leg and thigh. They developed an algorithm for estimating the uniaxial angles of the joints. All modules contained two accelerometers and one gyroscope [23]. For inertial data processing in the measurement system, some authors used the Butterworth low-pass filter [24]. In our test, we used optical Vicon Nexus system (Vicon Motion Systems Ltd, Yarnton, UK) to validate each sensor before the experiment [25]. The 20-s stand test was used to calculate drift parameters and orientation of each sensor with the Vicon system. This system has protocols and, during gait, we treated it as a gold standard. It was used as a truth-reference and we could compare both signal results. The IMU data were recorded in a laptop. It operates independently of the Vicon system. Data were recorded in independent systems. They were post-processed separately.

### 2.2. Exoskeleton of the Lower Limb with Hydraulic Actuators Model

**Figure 2:**
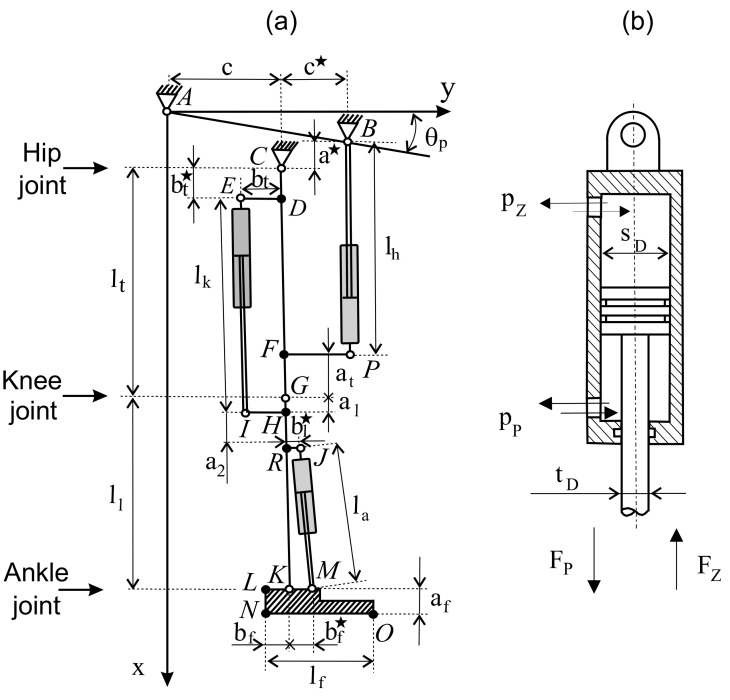
Structural diagram with parameters of the lower extremities’ hydraulic exoskeleton model (**a**) and cross-section of the double-acting hydraulic cylinder (**b**).

The kinematic diagram of the experimental model of the lower limb exoskeleton with hydraulic actuators is presented in Figure 2. To support the movement of the lower limb, three double-acting hydraulic actuators were proposed. The first actuator is responsible for the angle in the hip joint. The second, located in the rear part of the limb (in the sagittal plane), is responsible for the angle in the knee joint. Its characteristic feature is the lower catch, located below the knee joint. The role of the last third servomotor is to determine the right angle in the ankle joint. The mechanical structure of a proposed exoskeleton model is pseudo-anthropomorphic. The exoskeleton model is similar to a human's leg, but does not include all of DoF. A pseudo-anthropomorphic exoskeleton model has three DoF per leg: the hip, the knee, and 1 degree at the ankle. Hip abduction/adduction and internal/external rotation are in a passive joint. Ankle adduction/abduction is also passive. Hydraulic actuators are designed along with the lower limb segments.

The coordinates of the characteristic points, in the adopted system, in the starting position (as in Figure 2a) were calculated as:

(2) $$\begin{array}{c} A=(0,0)\\ B=\left(\left(c+c^{*}\right)\mathrm{tg}\theta_{p},c+c^{*}\right)\\ C=\left(\left(c+c^{*}\right)\mathrm{tg}\theta_{p}+a^{*},c\right)\\ D_{0}=\left(\left(c+c^{*}\right)\mathrm{tg}\theta_{p}+a^{*}+b_{t}^{*},c\right)\\ E_{0}=\left(\left(c+c^{*}\right)\mathrm{tg}\theta_{p}+a^{*}+b_{t}^{*},c-b_{t}\right)\\ F_{0}=\left(\left(c+c^{*}\right)\mathrm{tg}\theta_{p}+a^{*}+l_{t}-a_{t},c\right)\\ G_{0}=\left(\left(c+c^{*}\right)\mathrm{tg}\theta_{p}+a^{*}+l_{t},c\right)\\ H_{0}=\left(\left(c+c^{*}\right)\mathrm{tg}\theta_{p}+a^{*}+l_{t}+a_{l},c\right)\\ I_{0}=\left(\left(c+c^{*}\right)\mathrm{tg}\theta_{p}+a^{*}+l_{t}+a_{l},c-b_{t}\right)\\ J_{0}=\left(\left(c+c^{*}\right)\mathrm{tg}\theta_{p}+a^{*}+l_{t}+a_{l}+b_{l}^{*},c+b_{l}^{*}\right)\\ K_{0}=\left(\left(c+c^{*}\right)\mathrm{tg}\theta_{p}+a^{*}+l_{t}+l_{l},c\right)\\ L_{0}=\left(\left(c+c^{*}\right)\mathrm{tg}\theta_{p}+a^{*}+l_{t}+l_{l},c-b_{f}\right)\\ M_{0}=\left(\left(c+c^{*}\right)\mathrm{tg}\theta_{p}+a^{*}+l_{t}+a_{l}+b_{l}^{*},c+b_{f}^{*}\right)\\ N_{0}=\left(\left(c+c^{*}\right)\mathrm{tg}\theta_{p}+a^{*}+l_{t}+l_{l}+l_{a},c-b_{f}\right)\\ \left.O_{0}=\left(\left(c+c^{*}\right)\mathrm{tg}\theta_{p}+a^{*}+l_{t}+l_{l}+l_{a},c+l_{f}\right)-b_{f}\right)\\ P_{0}=\left(\left(c+c^{*}\right)\mathrm{tg}\theta_{p}+a^{*}+l_{t}-a_{t},c+c^{*}\right)\\ R_{0}=\left(\left(c+c^{*}\right)\mathrm{tg}\theta_{p}+a^{*}+l_{t}+a_{2},c\right) \end{array}$$

The coordinates of the Equation (2) depend on the anthropometric parameters that can be measured individually or based on data proposed by Winter and Hof [26,27,28]. The constant distances between points, necessary in further calculations, were determined from the relationship below.

(3) $$\begin{array}{cc} \; & \bar{AB}=\left(c+c^{*}\right)\sqrt{1+{\mathrm{tg}}^{2}\theta_{p}}\\ \; & \bar{CA}=\sqrt{c^{2}+{\left(\left(c+c^{*}\right)\mathrm{tg}\theta_{p}+a^{*}\right)}^{2}}\\ \; & \bar{CB}=\sqrt{{\left(c^{*}\right)}^{2}+{\left(a^{*}\right)}^{2}}\\ \; & \bar{CE}=\sqrt{{\left(b_{t}^{*}\right)}^{2}+b_{t}^{2}}\\ \; & \bar{CP}=\sqrt{{\left(c^{*}\right)}^{2}+{\left(l_{t}-a_{t}\right)}^{2}}\\ \; & \bar{IG}=\sqrt{b_{t}^{2}+a_{l}^{2}}\\ \; & \bar{JG}=\sqrt{{\left(b_{l}^{*}\right)}^{2}+{\left(a_{l}+a_{2}\right)}^{2}}\\ \; & \bar{EG}=\sqrt{b_{t}^{2}+{\left(l_{t}-a_{1}\right)}^{2}}\\ \; & \bar{JK}=\sqrt{{\left(b_{l}^{*}\right)}^{2}+{\left(l_{l}-a_{l}-a_{2}\right)}^{2}}\\ \; & \bar{KM}=b_{f}^{*} \end{array}$$

The next step was to select actuators for the exoskeleton. The problem of actuation design is to find an actuator (minimum length, diameter, and stroke). Bi-directional linear hydraulic actuators (called double-acting cylinders) with a one-sided piston rod were proposed as the actuators. In this type of actuator, the working fluid acts alternately on both sides of the piston. In the hydraulic exoskeletons, it is best to have double-acting cylinders because they move in two directions (flexion/extension). Moreover, this kind of actuator is faster, stronger, and uses less energy than single-acting cylinders. The actuator diagram of the hydraulic cylinder is shown in Figure 2b. The maximum value of the bending force F_maxZ_ and the straightening force F_maxP_, as a function of pressure *p*, actuator bore diameter *s_D_*, and diameter of the cylinder working chamber *t_D_*, were determined from the formula below.

(4) $$F_{maxZ}=p\frac{\pi {\left(s_{D}\right)}^{2}}{4},\;F_{maxP}=p\frac{\pi \left(s_{D}^{2}-t_{D}^{2}\right)}{4}$$

An actuator with a larger diameter produces more force and joint torque. However, it will require larger volumetric displacements for a given angular motion. Based on Equation (2), the length of the hydraulic cylinders in the starting position was defined below.

(5) $$\begin{array}{l} l_{h0}=a^{*}+l_{t}-a_{t}\\ l_{k0}=l_{t}-b_{t}^{*}+a_{l}\\ l_{a0}=\sqrt{{\left(b_{f}^{*}-b_{l}^{*}\right)}^{2}+{\left(l_{l}-a_{l}-b_{l}^{*}\right)}^{2}} \end{array}$$

Based on the dimension of the actuator, it is possible to calculate the location of the actuator end-points on two links. It is very important that the actuator action lines must not pass through the joint. To determine the location of characteristic points, directional cosine matrices were used [29]:

(6) $$M_{h}=\left[\begin{array}{cc} \cos\theta_{h} & -\sin\theta_{h}\\ \sin\theta_{h} & \cos\theta_{h} \end{array}\right],\;M_{k}=\left[\begin{array}{cc} \cos\theta_{k} & \sin\theta_{k}\\ -\sin\theta_{k} & \cos\theta_{k} \end{array}\right],\;M_{a}=\left[\begin{array}{cc} \cos\theta_{a} & \sin\theta_{a}\\ -\sin\theta_{a} & \cos\theta_{a} \end{array}\right]$$

The coordinates of the characteristic points of the hydraulic exoskeleton of the lower limbs, as a function of angles (*θ_h_, θ_k_, θ_a_*), were determined.

(7) $$\begin{array}{cc} \; & A=\left[\begin{array}{c} 0\\ 0 \end{array}\right]\\ \; & B=\left[\begin{array}{c} \left(c+c^{*}\right)\mathrm{tg}\theta_{p}\\ c+c^{*} \end{array}\right]\\ \; & C=\left[\begin{array}{c} a^{*}+\left(c+c^{*}\right)\mathrm{tg}\theta_{p}\\ c \end{array}\right]\\ \; & D=M_{h}\left[\begin{array}{c} b_{t}^{2}\\ 0 \end{array}\right]+C\\ \; & E=M_{h}\left[\begin{array}{c} b_{t}^{2}\\ -b_{t} \end{array}\right]+C\\ \; & F=M_{h}\left[\begin{array}{c} l_{t}-a_{t}\\ 0 \end{array}\right]+C\\ \; & G=M_{h}\left[\begin{array}{c} l_{t}\\ 0 \end{array}\right]+C\\ \; & H=M_{h}\left(M_{k}\left[\begin{array}{c} a_{l}\\ 0 \end{array}\right]+\left[\begin{array}{c} l_{t}\\ 0 \end{array}\right]\right)+C\\ \; & I=M_{h}\left(M_{k}\left[\begin{array}{c} a_{l}\\ -b_{t} \end{array}\right]+\left[\begin{array}{c} l_{t}\\ 0 \end{array}\right]\right)+C\\ \; & J=M_{h}\left(M_{k}\left[\begin{array}{c} a_{1}+a_{2}\\ b_{l}^{*} \end{array}\right]+\left[\begin{array}{c} l_{t}\\ 0 \end{array}\right]\right)+C\\ \; & K=M_{h}\left(M_{k}\left[\begin{array}{c} l_{l}\\ 0 \end{array}\right]+\left[\begin{array}{c} l_{t}\\ 0 \end{array}\right]\right)+C\\ \; & L=M_{h}\left(M_{k}\left(M_{a}\left[\begin{array}{c} 0\\ -b_{f} \end{array}\right]+\left[\begin{array}{c} l_{l}\\ 0 \end{array}\right]\right)\right)+C\\ \; & M=M_{h}\left(M_{k}\left(M_{a}\left[\begin{array}{c} 0\\ b_{f}^{*} \end{array}\right]+\left[\begin{array}{c} l_{l}\\ 0 \end{array}\right]\right)+\left[\begin{array}{c} l_{t}\\ 0 \end{array}\right]\right)+C\\ \; & N=M_{h}\left(M_{k}\left(M_{a}\left[\begin{array}{c} a_{f}\\ -b_{f} \end{array}\right]+\left[\begin{array}{c} l_{l}\\ 0 \end{array}\right]\right)+\left[\begin{array}{c} l_{t}\\ 0 \end{array}\right]\right)+C\\ \; & O=M_{h}\left(M_{k}\left(M_{a}\left[\begin{array}{c} a_{f}\\ l_{f}-b_{f} \end{array}\right]+\left[\begin{array}{c} l_{l}\\ 0 \end{array}\right]\right)+\left[\begin{array}{c} l_{t}\\ 0 \end{array}\right]\right)+C\\ \; & P=M_{h}\left[\begin{array}{c} l_{t}-a_{t}\\ c^{*} \end{array}\right]+C\\ \; & R=M_{h}\left(M_{k}\left[\begin{array}{c} a_{l}+a_{2}\\ 0 \end{array}\right]+\left[\begin{array}{c} l_{t}\\ 0 \end{array}\right]\right)+C \end{array}$$

From the derived Equation (7), the change in piston rod length from the starting position was determined, and the model is shown in Figure 2.

(8) $$\begin{array}{c} l_{h}\left(\theta_{h}\right)=\bar{BP}=\sqrt{{\left(P\left(1,1\right)-B\left(1,1\right)\right)}^{2}+{\left(P\left(2,1\right)-B\left(2,1\right)\right)}^{2}}-l_{h0}\\ l_{k}\left(\theta_{k}\right)=\bar{EI}=\sqrt{{\left(I\left(1,1\right)-E\left(1,1\right)\right)}^{2}+{\left(I\left(2,1\right)-E\left(2,1\right)\right)}^{2}}-l_{k0}\\ l_{a}\left(\theta_{a}\right)=\bar{JM}=\sqrt{{\left(M\left(1,1\right)-J\left(1,1\right)\right)}^{2}+{\left(M\left(2,1\right)-J\left(2,1\right)\right)}^{2}}-l_{a0}\\ \varepsilon_{1h}=\frac{l_{h0}-l_{h}\left(\theta_{h}\right)}{l_{h0}}\\ \varepsilon_{1k}=\frac{l_{k0}-l_{k}\left(\theta_{k}\right)}{l_{k0}}\\ \varepsilon_{1a}=\frac{l_{a0}-l_{a}\left(\theta_{a}\right)}{l_{a0}} \end{array}$$

The displacement of the actuator piston allows us to obtain the required bending moments *τ_maxF_* and straightening individual joints *τ_maxE_*, whose maximum values were determined as:

(9) $$\begin{array}{cc} \; & \tau_{h_{\max E}}=F_{\mathrm{maxp}}{\vec{r}}_{h}\times \frac{l_{h}\left(\theta_{h}\right)}{l_{h0}},\;\tau_{h_{\max F}}=F_{\max Z}{\vec{r}}_{h}\times \frac{l_{h}\left(\theta_{h}\right)}{l_{h0}}\\ \; & \tau_{k_{\max}E}=F_{\max P}{\vec{r}}_{k}\times \frac{l_{k}\left(\theta_{k}\right)}{l_{k0}},\;\tau_{k_{\max F}}=F_{\max Z}{\vec{r}}_{k}\times \frac{l_{k}\left(\theta_{k}\right)}{l_{k0}}\\ \; & \tau_{a_{\max E}}=F_{\max P}{\vec{r}}_{a}\times \frac{l_{a}\left(\theta_{a}\right)}{l_{a0}},\;\tau_{a_{\max}F}=F_{\max}{\vec{r}}_{a}\times \frac{l_{a}\left(\theta_{a}\right)}{l_{a0}} \end{array}$$

where *r* is the distance of the actuators from the joint axis. It is clear from the geometrical Equation (9) that the distance of the actuator from the joint has a decisive influence on the generated moment. The further the actuator is placed, the greater the torque that can be obtained. Similarly, larger diameter cylinders can generate greater torque, but this requires more working hydraulic fluid. The key thing is to select the appropriate actuator type (diameter, minimum and maximum length, and fixing at characteristic points B, P, E, I, J, and M). The cylinders should provide a full range of angular movement for each joint. At this point, attention should be paid to the method of mounting the cylinders (piston rod position) shown in Figure 2. At constant pressure, greater strength will be obtained when straightening the hip, knee, and plantar bending in the case of the ankle.

**Figure 3:**
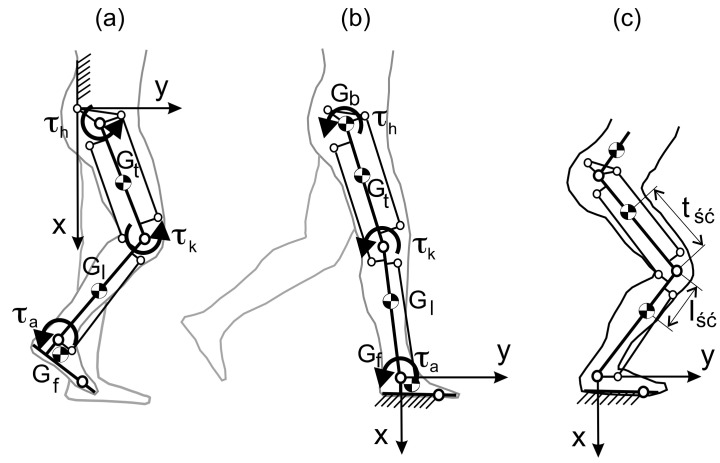
Hydraulic exoskeleton model of the lower limb in the sagittal plane in the transfer phase (**a**), in the support phase (**b**), and in the squat phase (**c**).

The operation of the exoskeleton has two phases: the transfer phase and the support phase, and models are shown in Figure 3a,b. When analyzing the transfer phase, it is assumed that the upper part of the human body with the device (pelvis, torso, exoskeleton control, and power supply) is immobilized. A detailed mathematical model of a limb equipped with an exoskeleton with a hydraulic actuator, including the foot model in the support and transfer phase, can be found in Reference [30].

## 3. Results

**Figure 4:**
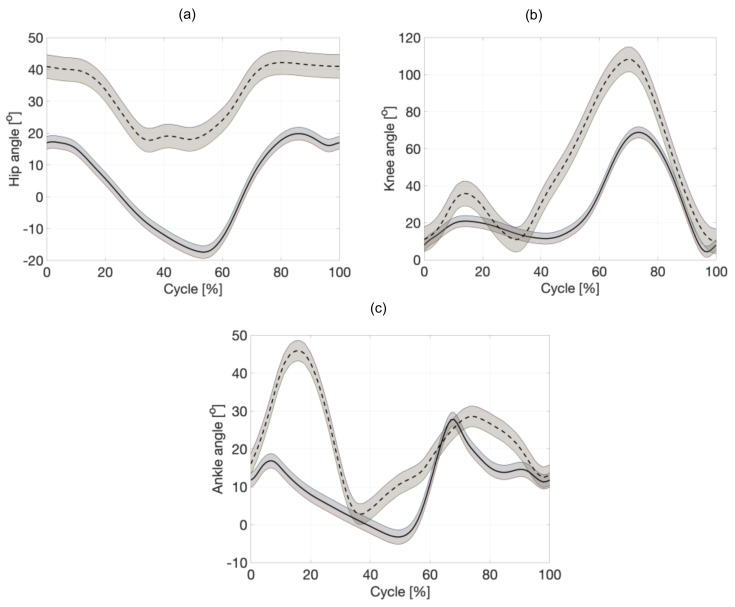
Anteroposterior joint angles during preferred walking (—) and running (---). The percentage of the gait cycle is shown on the *x*-axis. (**a**) hip, (**b**) knee, and (**c**) ankle.

**Figure 5:**
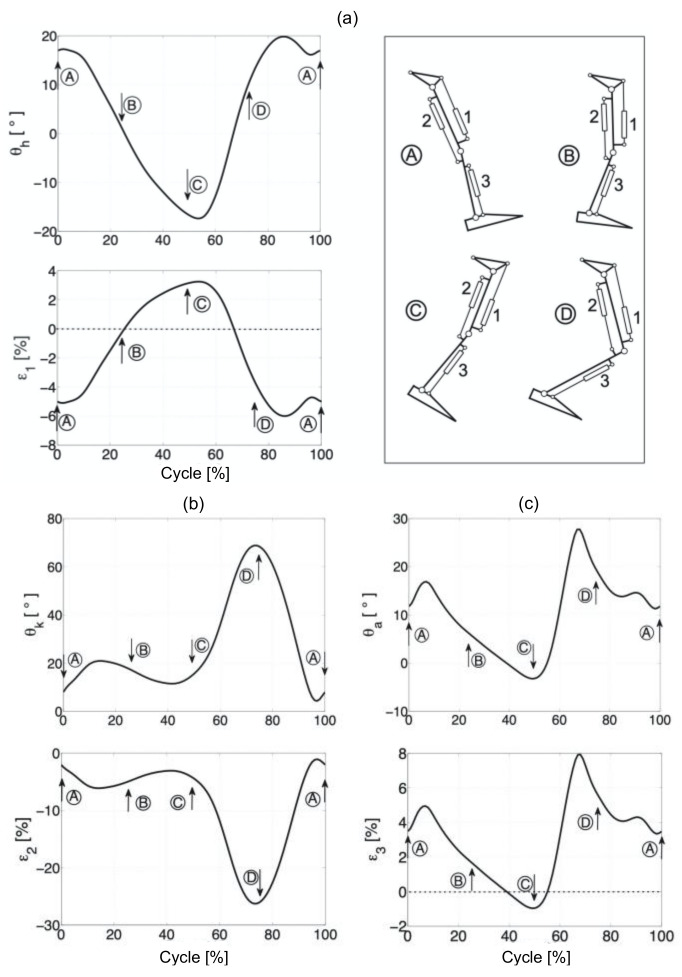
The angles in the joints and length of the hydraulic exoskeleton actuators, while walking: Hip joint (**a**), knee joint (**b**), and ankle joint (**c**).

**Figure 6:**
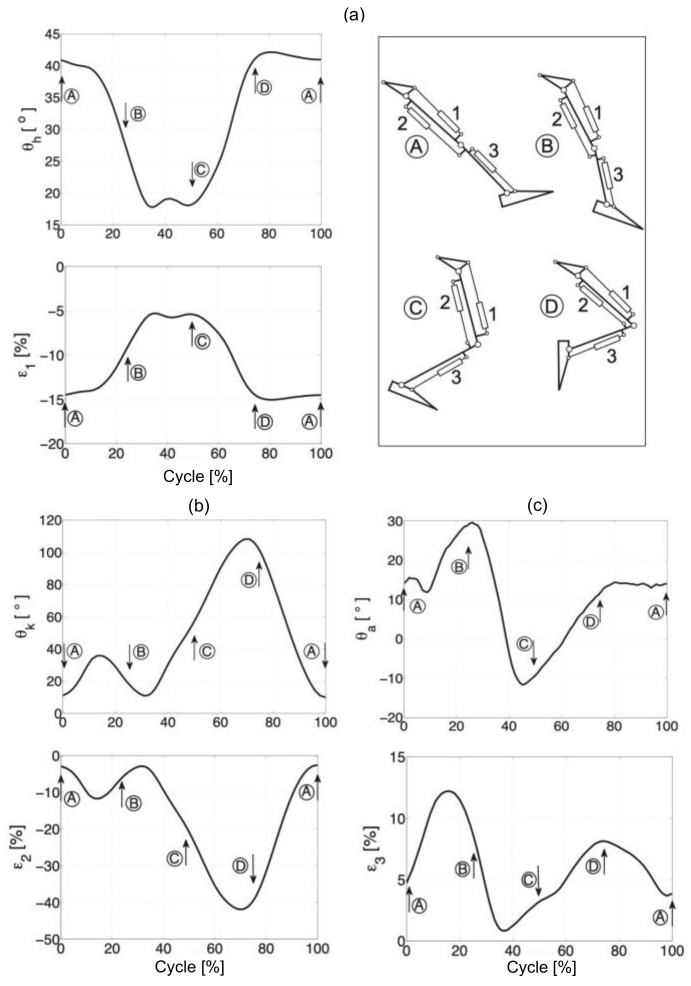
The angles in the joints and length of the hydraulic exoskeleton actuators, while running: Hip joint (**a**), knee joint (**b**), and ankle joint (**c**). A, B, C and D-exoskeleton positions presented on the top right side.

Data are presented graphically so that time is normalized to 100% of the cycle (Figure 4). Figure 5 and Figure 6 show the change in the angles of individual joints and the length of the hydraulic exoskeleton actuators in free walking and while running. The actuator piston length was determined as half the distance between the cylinder attachment points in the initial position. Individual actuators are marked in the drawing with numbers from 1 to 3. The hydraulic exoskeleton of each lower limb has three degrees of freedom in the sagittal plane.

In the proposed model, the mass and location of the centres of gravity of the thigh, lower leg, and foot are known, along with the moments of inertia relative to the axis perpendicular to the sagittal plane. During the transfer phase, the foot rotation around the axis passing through the ankle is skipped. The equation of motion in the transfer phase can, therefore, be written as:

(10) $$H\left(\theta \right)\ddot{\theta }+C\left(\theta ,\dot{\theta }\right)\dot{\theta }+G\left(\theta \right)=\tau$$

where θ*(t)* is a vector of joint variables, the vector of individual angles is $\theta ={\left[\theta_{h},\theta_{k},\theta_{a}\right]}^{T}$, and the vector of angular velocities of individual joints is $\dot{\theta }={\left[{\dot{\theta }}_{h},{\dot{\theta }}_{k},{\dot{\theta }}_{a}\right]}^{T}$. The matrix of required moments in individual joints from actuators is $\tau ={\left[\tau_{h},\tau_{k},\tau_{a}\right]}^{T}$. The symmetrical matrix determining the inertia of the limb together with the exoskeleton is marked as **H**(*θ*). The velocity coupling matrix is $C{\left(\theta ,\dot{\theta }\right)}^{T}$, and the matrix $G\left(\theta \right)={\left[\theta_{h},\theta_{k},\theta_{a}\right]}^{T}\;$ describes the gravitational forces acting on individual limb segments. The moments in individual joints τ are the sum of the moments coming from the user's muscles $\tau_{u}$ and the actuators $\tau_{S}$. They are calculated according to the Equation (8), where, in the transfer phase, the value of the moment $\tau_{a}$ is assumed to be 0. In this model, the phenomenon of friction is not taken into account.

**Figure 7:**
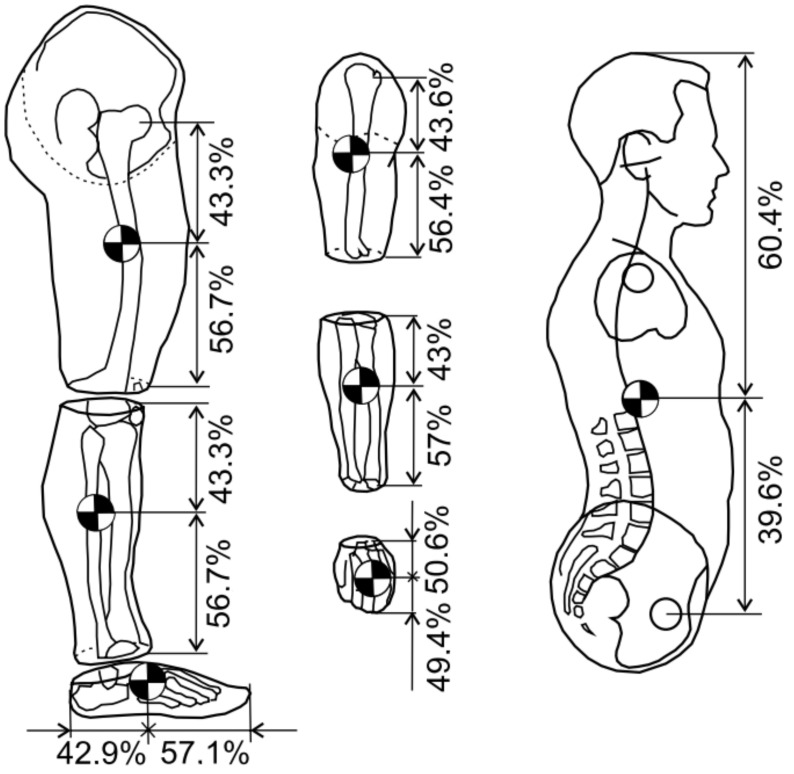
Location of the centres of gravity of individual segments of the human body.

The vector of articulated variables has three components, so the dimension of the generalized force vector τ(t) is also three. Unlike the previous model, the mass of the upper body and exoskeleton, with a load on the user’s back, has been simplified to a mass concentrated in the middle of the hip at one point. The equation of motion in the support phase takes the same form as the Equation (8). The moment in the ankle joint $\tau_{a}$ arises from the user’s muscles, while, in the knee joint, it is the sum of the moment from the muscles $\tau_{u}$ and the actuator $\tau_{k}$. The location of the centres of gravity of individual body parts is shown in Figure 7.

Mass values of individual segments of the human body are based on Reference [13]. An even distribution of gravity is assumed for each limb. The masses of individual limb segments were determined on the basis of anthropometric tests, and their values are, respectively, *m_t_* = 8 kg (tight), *m_l_* = 4.52 kg (shank), and *m_b_* = 27.12 + 25 = 43.12 kg, where the value of 25 kg corresponds to the mass of the load and exoskeleton per one leg [31]. The position of the centre of mass of the thigh, 1 and 2 actuators, relative to the knee joint, can be defined as:

(11) $$\begin{array}{c} x_{Gc}=\frac{\sum^{\;}m_{i}x_{i}}{m_{i}}=\frac{m_{t}x_{t}+m_{1}x_{1}+m_{2}x_{2}}{m_{t}+m_{1}+m_{2}},\\ y_{Gc}=\frac{\sum^{\;}m_{i}y_{i}}{m_{i}}=\frac{m_{t}y_{t}+m_{1}y_{1}+m_{2}y_{2}}{m_{t}+m_{1}+m_{2}}. \end{array}$$

whereas the shank and actuator 3 centre of mass relative to the ankle joint is calculated as:

(12) $$\begin{array}{c} x_{Kc}=\frac{m_{l}x_{l}+m_{3}x_{3}}{m_{l}+m_{3}},\\ y_{Kc}=\frac{m_{l}y_{l}+m_{3}y_{3}}{m_{l}+m_{3}}. \end{array}$$

**Figure 8:**
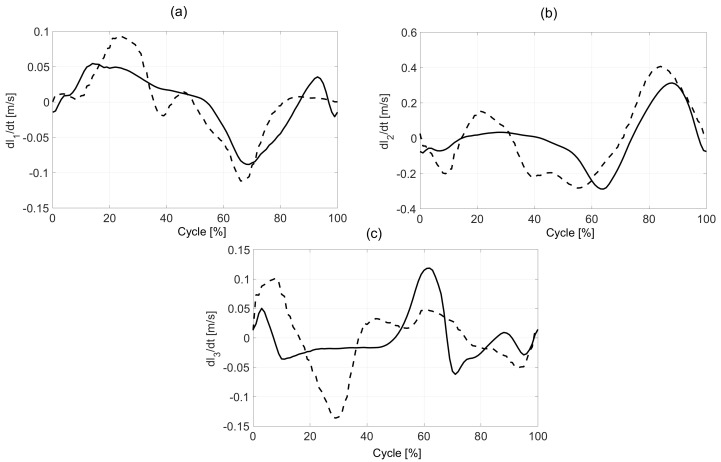
The actuators velocities during walking (—) and running (---): Hip actuator (**a**), knee actuator (**b**), and ankle actuator (**c**).

Figure 8 presents the velocity of changing the length of the actuators during walking and running. It was observed that the maximal values during walking 0.3 m/s and running 0.4 m/s are in the second actuator (knee joint) during a swing phase. For the hip, the maximal value is 0.12 m/s for walking and 0.09 m/s for running.

## 4. Discussion

In this paper, we presented the kinematics of exoskeleton hydraulic actuators when walking and running. The main aim of the letter was to present a proposal for designing an exoskeleton with the hydraulic actuators. We built a geometrical model of a hydraulic exoskeleton based on anthropometrical parameters. It may be used in the kinematic and kinetic simulation of human movement. The angles during walking of joints were calculated using IMUs. The use of IMUs to calculate angles is suitable for investigators because of the limited need for equipment. Angle values obtained can be used to determine the operating parameters of exoskeletons. Next, by implementing these parameters in the model, it is possible to analyze how actuators should work. Determination of kinematic parameters enables the selection of hydraulic actuators and geometric parameters.

In our model, we did not normalize the joint angles to human height. Dempster has suggested scaling data to a human size to adjust for various participant anthropometrics [28]. We decided to present our results without standardization. The purpose was to present data that are easily implemented to the exoskeleton model. The differences in anthropometrics between of our study participants may impact joint parameters and exoskeleton actuator velocities.

We examined preferred walking and running speeds. If the exoskeleton was to be used in the rehabilitation of people with mobility problems, such as cerebral palsy or stroke, a range of lower speeds of movement should be considered [2,21]. Future research should be focused on a walking speed range of 0.40 to 1.6 m/s. According literature, it is representative of the range of gait speeds in clinical research [31].

The current study was only conducted in healthy adults by using the IMU. Unfortunately, it does not give a picture of people with a walking disability [32,33]. Future research should include an analysis of the angular velocity range of the joints. Much work needs to be done to accurately design the detailed exoskeleton model, analyze its performance, and make a real model. To optimize the operation of the exoskeleton, it is also necessary to conduct parametric studies to select the best possible values of the design variables. Unique results were presented by Kańtoch who proposed developing smart clothing that integrates IMU sensors into garments [34]. Therefore, this solution is possible to monitor human activity, which can be used to calculate human position as a signal for exoskeletons.

Finally, it should be noted that the proposed kinematic model of the exoskeleton may be the basis for the real model and the analysis of gait dynamics, which will be presented in the next publication.
